# Identity, structure, and function of the mitochondrial permeability transition pore: controversies, consensus, recent advances, and future directions

**DOI:** 10.1038/s41418-023-01187-0

**Published:** 2023-07-17

**Authors:** Paolo Bernardi, Christoph Gerle, Andrew P. Halestrap, Elizabeth A. Jonas, Jason Karch, Nelli Mnatsakanyan, Evgeny Pavlov, Shey-Shing Sheu, Alexander A. Soukas

**Affiliations:** 1 Department of Biomedical Sciences, University of Padova, Padova, Italy; 2 Laboratory of Protein Crystallography, Institute for Protein Research, Osaka University, Suita, Japan; 3 School of Biochemistry and Bristol Heart Institute, University of Bristol, Bristol, UK; 4 Department of Internal Medicine, Section of Endocrinology, Yale University School of Medicine, New Haven, CT USA; 5 Department of Integrative Physiology and Biophysics, Baylor College of Medicine, Houston, TX USA; 6 Department of Cellular and Molecular Physiology, College of Medicine, Penn State University, State College, PA USA; 7 Department of Molecular Pathobiology, New York University, New York, NY USA; 8 Department of Medicine, Center for Translational Medicine, Sidney Kimmel Medical College, Thomas Jefferson University, Philadelphia, PA USA; 9 Department of Medicine, Diabetes Unit and Center for Genomic Medicine, Massachusetts General Hospital and Harvard Medical School, Boston, MA USA

**Keywords:** Cell biology, Diseases

## Abstract

The mitochondrial permeability transition (mPT) describes a Ca^2+^-dependent and cyclophilin D (CypD)-facilitated increase of inner mitochondrial membrane permeability that allows diffusion of molecules up to 1.5 kDa in size. It is mediated by a non-selective channel, the mitochondrial permeability transition pore (mPTP). Sustained mPTP opening causes mitochondrial swelling, which ruptures the outer mitochondrial membrane leading to subsequent apoptotic and necrotic cell death, and is implicated in a range of pathologies. However, transient mPTP opening at various sub-conductance states may contribute several physiological roles such as alterations in mitochondrial bioenergetics and rapid Ca^2+^ efflux. Since its discovery decades ago, intensive efforts have been made to identify the exact pore-forming structure of the mPT. Both the adenine nucleotide translocase (ANT) and, more recently, the mitochondrial F_1_F_O_ (F)-ATP synthase dimers, monomers or c-subunit ring alone have been implicated. Here we share the insights of several key investigators with different perspectives who have pioneered mPT research. We critically assess proposed models for the molecular identity of the mPTP and the mechanisms underlying its opposing roles in the life and death of cells. We provide in-depth insights into current controversies, seeking to achieve a degree of consensus that will stimulate future innovative research into the nature and role of the mPTP.

## Facts

- The mPTP is a non-selective channel, permeable to solutes of <1.5 kDa that forms in the inner mitochondrial membrane under conditions of elevated matrix [Ca^2+^] and oxidative stress.
- Opening of mPTP may be prolonged or transient and there may be substates with different conductance levels.
- The precise molecular identity of the mPTP remains uncertain but strong evidence implicates both the mitochondrial F_1_F_O_ (F)-ATP synthase and the adenine nucleotide translocase (ANT), with matrix cyclophilin D (CypD) facilitating the transition to the pore-forming conformation.
- Prolonged mPTP opening is pathological leading to cell death such as occurs in ischemia/reperfusion injury while transient pore opening may occur physiologically and regulate mitochondrial bioenergetics, cellular metabolism, Ca^2+^ homeostasis and ROS production.

## Open questions

- What conformational changes occur in the F-ATP synthase or ANT to cause the mPTP channel(s) to form? Do both the F-ATP synthase and the ANT form distinct channels with different conductances or is the mPTP formed through an interaction of both within the proposed ATP synthasome? What are their separate and/or overlapping roles in mPTP formation? Are there additional mPTP channels beside F-ATP synthase and ANT?
- What is the binding site for matrix Ca^2+^ that triggers pore opening and how do known modulators of mPTP formation, such as oxidative stress, adenine nucleotides, phosphate and CypD, exert their effects?
- How to connect the in vitro molecular and structural studies to the in vivo functional studies under physiological and pathological conditions?
- What events determine the transition of a very small fraction of ANT and F-ATP synthase to act as mPTP instead of performing their canonical role in ATP generation and transport? Does this minute fraction that acts as mPTP randomly occur or is it specifically regulated by signaling mechanisms in microdomains?
- How does mPTP change mitochondrial structure and function? Conversely, how do changes in mitochondrial structure lead to activation or inactivation of mPTP?
- What is the role of mPTP in development, metabolism, aging, and degenerative diseases?

## Introduction

The mitochondrial permeability transition (mPT) describes a highly-reproducible, Ca^2+^-dependent increase of inner mitochondrial membrane permeability to ions and solutes [1–5]. It is mediated by a putative channel, the mitochondrial permeability transition pore (mPTP), which has also been recorded electro-physiologically and called the mitochondrial mega-channel or multi-conductance channel [6, 7]. Depending on the specific conductance state, mPTP properties may vary from a small non-selective ion channel to a large pore that allows diffusion of molecules up to 1.5 kDa in size. Sustained mPTP opening causes disruption of mitochondrial energy metabolism and necrotic cell death [8]. Furthermore, mPTP activation can lead to osmotic dysregulation of mitochondria leading to mitochondrial swelling [1–4]. This may rupture the outer mitochondrial membrane causing release of cytochrome *c* from the intermembrane space into the cytosol which will initiate apoptotic cell death if cellular ATP levels can be maintained [9]. These mPTP-mediated cell death mechanisms play a key role in the pathogenesis of numerous, aging-associated human diseases including, but not limited to, ischemic heart and brain disease, liver and kidney failure, cancer, and degenerative diseases [10–13]. However, transient mPTP opening, perhaps involving different sub-conductance states, may exert several physiological roles such as transient rapid alterations in mitochondrial bioenergetics, reactive oxygen species (ROS) production, Ca^2+^ efflux and metabolic regulation [14–18]. High levels of matrix Ca^2+^, inorganic phosphate, cyclophilin D (CypD) and oxidative stress activate mPTP opening, while divalent and trivalent cations such as Mg^2+^, Ba^2+^, Gd^3+^, in addition to H^+^, adenine nucleotides and cyclosporine A (CsA), inhibit the opening [19–21].

The exact pore-forming structure of the mPT remains controversial. The voltage-dependent anion channel (VDAC) [22], proapoptotic Bcl-2 family members (Bax and Bak) [9, 23–25], the phosphate carrier (PiC) [26] and the translocator protein (TSPO) [27] had been proposed to contribute to the channel of the mPT. However, subsequent studies have shown that PiC more likely regulates the probability of mPTP opening, rather than constituting the CsA-sensitive pore of mPT, [28–33] while VDAC together with pro-apoptotic Bcl-2 molecules likely facilitate permeation across the outer membrane or modulate interactions between the inner and outer mitochondrial membranes that can influence mPT activity. None of these proteins serves as the pore (channel) of mPT, and the effect of ligands of TSPO can probably be explained by their binding to subunit OSCP of ATP synthase [34]. The adenine nucleotide translocator (ANT) [35] may contribute a smaller conductance activity than that of the largest amplitude conductance of mPTP and may also play a role in the opening of mPTP [36]. Thus, ANT genetic deletion alone greatly impairs but does not completely eliminate CypD-sensitive mPTP. Most recently, mitochondrial F_1_F_O_ (F)-ATP synthase dimers [37–41], monomers [42] or c-subunit ring [33, 42–47] have been suggested to be the main CypD-regulated mPT channel. However, it is likely that not all contributing channel-forming complexes have yet been identified. Nevertheless, the recognition of key roles for both the ANT and F-ATP synthase is intriguing considering that both are critical for the synthesis of ATP in the final step of oxidative phosphorylation and thus are essential for efficient cell metabolism and survival.

The heated debate surrounding the identity, structure, and two opposite functional roles (life versus death) of the mPTP have been ongoing for many years. In recent years the debate has intensified as a result of the reported cryoEM-based atomic models of mitochondrial and other F-ATP synthases [33–35, 38–43, 45]. In addition, there is now a better appreciation of the potential pitfalls in protein purification or mitoplast preparation for recording single channel activities of the mPTP in vitro. Thus, it is timely that the different perspectives of several key investigators who have pioneered mPT research are brought together in a critical assessment of proposed models for the molecular identity of the mPTP and the mechanisms underlying its opposing roles in the life and death of cells. The pathophysiological significance of mPTP will be exemplified by the well-established ischemia-reperfusion injury model in heart and the newly studied aging process. The overall aim is to provide in-depth insights into current controversies on the nature and role of the mPTP with the hope of achieving a certain degree of consensus based on currently available data that will stimulate future innovative research.

## The mPTP structure

### Challenges of studying mPTP in vitro

One of the approaches used to demonstrate the existence of an ion channel is to record its biophysical characteristics, such as single channel conductance, with electrophysiological techniques. This has been reported for the ion channels that are involved in causing mPT [6, 7]. Despite high fidelity characterization of mPT channel activity by mitoplast patch-clamp, investigations have been severely impacted by the experimental difficulties of probing the molecular identity underlying the measured currents. In particular, standard practices of injecting cDNA of plasmalemmal channel forming proteins into *Xenopus* oocytes or transfecting model cultured cells for patch-clamp measurements do not work for multi-subunit mitochondrial membrane proteins such as the F-ATP synthase. Likewise, the technical difficulties of isolating and reconstituting multi-subunit mitochondrial membrane complexes into lipid bilayers for electrophysiological measurements have hindered our ability to unequivocally pin down the molecular identity or single molecule properties of the mPTP. Furthermore, as discussed further below, for mPTP opening it is likely that only a very small fraction of the relevant protein is present in the distinct pore-forming conformation [48, 49]. Consequently, the mPTP field suffers from the lack of advanced in vitro structure-function investigations common to other membrane proteins such as K^+^ channels or G-protein coupled receptors (GPCRs). High-level in vitro structure-function studies are an essential basis for the development of effective small molecule drug compounds in the treatment of mPTP related pathologies [50].

**Fig. 1:**
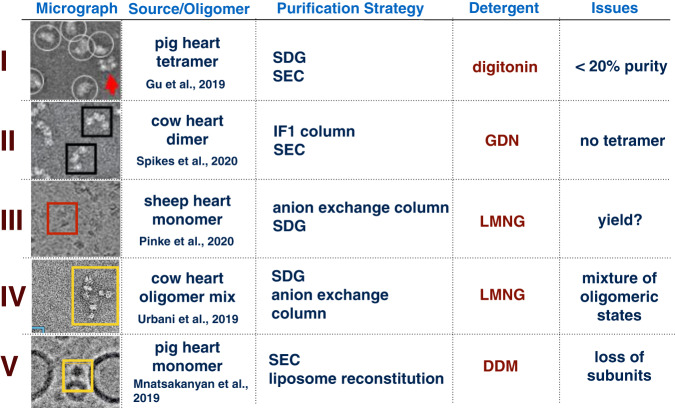
State-of-the-art in mammalian F-ATP synthase purification. Isolation of mammalian F-ATP synthase from mitochondria is the basis of any in vitro experiment that examines its role as the mPTP. Though difficulties remain, new purification strategies and use of novel detergents have allowed significant progress. Roman numerals I to V indicate selected published examples from refs. [41, 52, 53, 67] and [42], respectively. Micrographs are shown at comparable magnification. SDG sucrose density gradient, SEC size exclusion chromatography.

Mitochondrial F-ATP synthase has been proposed as an important molecular entity underlying mPTP currents detected by patch clamped mitoplasts [37]. Mitochondrial F-ATP synthase is an integral membrane supercomplex central to cellular bioenergetics that forms dynamic rows of F-ATP synthase dimers (MW of >1.2 mDa, ~56 subunits for each dimer) which shape the inner mitochondrial membrane by inducing strong positive membrane curvature [51]. The mammalian F-ATP synthase dimer is highly fragile and the successful preparation of the intact dimer or tetramer supercomplex has thus far only been possible by using the very mild detergent digitonin [52] or the second generation non-ionic detergents glyco-diosgenin (GDN) [53] and lauryl maltose neopentyl glycol (LMNG) [41, 54] that were both originally developed for GPCR studies [55] (Fig. 1). In contrast, liposome reconstitution of the mammalian F-ATP synthase using conventional detergents such as decylmaltoside or dodecylmaltoside (DDM) has been reported only for its monomeric form [42, 56] (Fig. 1). Structural studies of intact F-ATP synthase by X-ray crystallography, have never succeeded because the F-ATP synthase is resistant to 3D crystal formation, irrespective of its biological source. The recent “resolution revolution” [57] in cryo-EM enabled the structure determination of F-ATP synthases thus allowing, for the first time, the building of atomic models at the amino acid side chain level. Such structural investigations of mammalian and yeast mitochondrial F-ATP synthases still lag behind those of chloroplast or bacterial F-ATP synthases [58, 59]. However, recent studies show an amazing structural diversity for the oligomeric mitochondrial F-ATP synthase from various organisms [60–64] that stands in stark contrast to the relative uniformity of strictly monomeric chloroplast and bacterial F-ATP synthases.

The fragility of mammalian dimeric/oligomeric mitochondrial F-ATP synthase during purification procedures has also imposed significant challenges in studying single channel activities by reconstituting the protein complex in lipid bilayer systems or patch clamping of giant unilamellar vesicles (GUVs). This problem is now resolved by the discovery that oligomeric mitochondrial F-ATP synthase can be auto inserted into pre-formed lipid bilayers if prepared at low concentration (~0.002%) of the high affinity detergent LMNG [41, 65, 66]. The use of LMNG or GDN has allowed the purification of relatively large amounts of a highly pure and stable mix of monomeric and oligomeric complexes of F-ATP synthase [41, 53, 67] (Fig. 1). Nevertheless, preparation of damage-free, pure, monomeric, dimeric, tetrameric and higher oligomeric mitochondrial F-ATP synthase still remains challenging.

The ANT is an additional candidate for the molecular entity underlying mPTP currents. Though considerably smaller in size as well as being structurally less diverse between species, the problems related to its in vitro investigation are similar to those of the F-ATP synthase; namely, the difficulties of isolating membrane proteins from the inner mitochondrial membrane while avoiding purification artifacts. This is illustrated for instance by the ongoing debate as to whether the physiologically relevant form of ANT is monomeric or dimeric [68–70].

When successful, in vitro structural studies of the candidate proteins such as F-ATP synthase should, hopefully, permit i) reliable atomic models of the mPTP; ii) visualization of the mPTP in an open state; iii) visualization of agonists, antagonists and inhibitors bound to the mPTP. However, these structural goals face the following challenge: the resolution of current cryo-EM density maps is still relatively far from the desired resolution of better than 2.0 Å for the transmembrane regions, leaving numerous ambiguities and impacting the description of bound small molecule effectors. The recent introduction of cold field emission guns and monochromator technology to cryo-EM [71, 72] and improved cryo-grid preparation might enhance cryo-EM density maps considerably in the near future. Another problem is that open state visualization could require the presence of a lipid bilayer and perhaps a transmembrane electric potential. Therefore, single particle approaches for proteoliposomes [42, 73] instead of detergent stabilized complexes might be necessary for open state visualization. Moreover, the seemingly low percentage of channel-active F-ATP synthase among the total of all mitochondrial F-ATP synthases in a given mitochondrion [49] is not compatible with single particle cryo-EM based structure determination because this approach requires the presence of many thousands of protein complexes in identical conformational states. This necessitates the identification of factors that allow channel activation for close to 100% of the membrane reconstituted F-ATP synthase complexes. In situ cryo-electron tomography of mitochondria after induction of mPTP opening [74] might represent one possible solution for many of the challenges described above, especially when combined with modern cryo focused-ion-beam milling [75]. However, the contrast diminishing exerted by the high density of matrix content poses a formidable challenge for in situ studies, effectively making efforts directed at in vitro studies of membrane reconstituted systems more readily tractable.

Among the many current problems of in vitro structure-function studies involving the F-ATP synthase perhaps the most pressing appears to be the lack of structures for yeast and human F-ATP synthase mutants that were already examined by in situ studies using electrophysiology or swelling assays. Establishment of purification procedures for the in vitro study of yeast and human F-ATP synthase mutants with altered mPTP behavior should therefore have a high priority. The now published structure of monomeric human F-ATP synthase isolated from HEK cells is a big step forward towards structural analysis of human F-ATP synthase mutants, which hopefully will become available soon [76]. However, a word of caution is required on the power of structural studies alone to resolve the molecular mechanism of channel gating. Despite the reporting of numerous high resolution structures of gap junction channels with similar conductance to full mPTP opening, the gating mechanism of these channels is still not understood and it appears that lipids of the embedding membrane are involved [77, 78].

Clearly, the problem of the molecular identity of the mPTP and the proper handling of mPTP forming protein complexes is still in the process of being solved. Nevertheless, it is worthwhile considering what kind of developments might be able to bridge the wide gap between in vitro experiments using highly reductionist molecular set-ups and what is known from studies of isolated mitochondria, cells and animal models of mPT functioning. Both electrophysiological recordings as well as visualization of purified F-ATP synthase complexes by cryo-EM are done under conditions that are far removed from those of mitochondria in vivo. Therefore, the future establishment of a “Synthetic Mitoplast” using perhaps GUVs reconstituted with a multitude of mitochondrial proteins, including oligomeric F-ATP synthase, and related molecular factors might be able to build a bridge between in vitro and in vivo studies. A “Synthetic Mitoplast”, if built from GUVs, would be in the sweet spot of being big enough to be used for electrophysiological measurements by patch clamp recordings and still small enough for structural investigation by cryo electron tomography (cryo-ET) or even in silico simulations. Similar to cryo-EM, computational biology has seen great progress in recent years and if supplied with sufficiently accurate experimental data could lead to in silico models that are able to simulate what molecular events are taking place during mPT, enabling insights that are currently out of experimental reach. Thus far the involvement of computational approaches in mPT research is rather limited [79] and consequently the potential for important contributions is large. Likewise, though some cryo-ET studies have been reported using correlated light microscopy [74] in situ cryo-ET on both isolated mitoplasts as well as a “Synthetic Mitoplast” are still in their infancy or even absent and thus very promising approaches for further development. For effectively connecting in vitro molecular approaches and in vivo studies ideally a research collaboration should be established that enables labs working on all levels including a dedicated effort to establish the “Synthetic Mitoplast” for addressing the many remaining riddles of mPT research.

In conclusion, further resolution of the molecular mechanisms of mPT will require solutions for the above-mentioned technical hurdles for the isolation of F-ATP synthase and other candidate proteins for mPTP, the efficient membrane reconstitution of mPTP candidates ideally into a “Synthetic Mitoplast”, and their characterization by electrophysiology and cryo-EM in combination with pharmacological manipulation.

### Multiple mechanisms of pore formation

A consensus is developing that the pore of mPT may be mediated by at least two molecular species. The first is the ANT, which can undergo a Ca^2+^-dependent transition to a channel [80] favored by atractyloside and inhibited by bongkrekate [3, 33, 81]. The second is the F-ATP synthase, which forms channels that display the key features of the mPTP after reconstitution in lipid bilayers and giant liposomes [41, 42]. The mechanisms through which a high-conductance channel originates from ANT and F-ATP synthase is under active investigation.

#### F-ATP synthase dimer

**Fig. 2:**
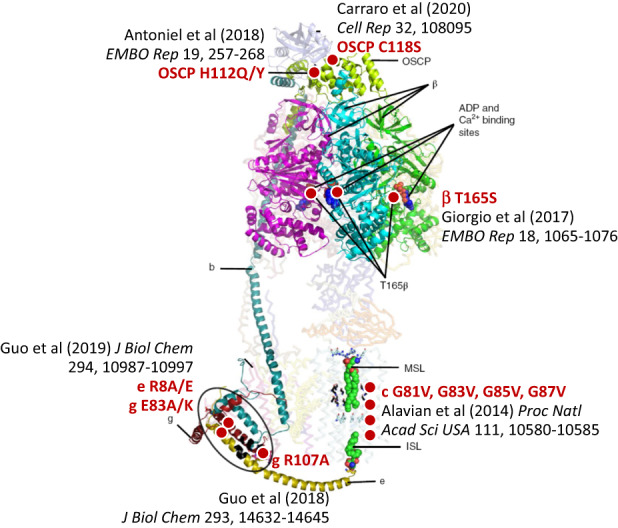
Point mutations of F-ATP synthase affecting its channel properties. The figure reports mutations of F-ATP synthase that affect specific features of the mPTP, i.e., conductance [47, 58], Ca^2+^-dependence [98], inhibition by H^+^ [279], sensitivity to glyoxals [280], and oxidants [281]. For details the reader is referred to the original publications indicated on the picture. Approximate positions of the mutations are indicated by red dots. The structure of F-ATP synthase used in the background is taken from ref. [67].

In the case of the F-ATP synthase, two non-mutually exclusive hypotheses have been proposed. In the first, channel formation would require enzyme dimers [37], which in higher eukaryotes are the building blocks of oligomers aligned in the inner membrane cristae ridges [82]. In the second, the channel would form within the c-ring of monomers after displacement of the lipid plug [47]. There is agreement that channel opening requires long-range conformational effects originating at the catalytic sites in the F_1_ sector after Ca^2+^ binding; and that these are transmitted to the inner membrane by peripheral stalk subunits OSCP, b, g, and e [81, 83–85]. There is an apparent discrepancy on whether a high-conductance channel can form within the ATP synthase enzyme monomers, which has been seen in highly purified F-ATP synthase preparations extracted with DDM [42] but not with LMNG [41]. It is possible that channel activity observed in monomers extracted with DDM may reveal a bona fide mPTP that has, however, lost the requirement for activation by Ca^2+^ because of partial removal of subunits e and g [42], which are preserved by LMNG [41]. In the intact system, Ca^2+^-dependent displacement of subunit e may be essential for channel activation, and this may require the cooperation of two monomers. It cannot be excluded that a channel may also form at the monomer-monomer interface. This might explain the existence of multiple conductance levels within the channel that are observed both in the native membrane [7] and in the reconstituted systems [41, 42]. Channel formation by F-ATP synthase is supported by a recent high-resolution structure of F-ATP synthase [67], which is consistent with the effects of a number of point mutations affecting specific channel features (Fig. 2). Additional discussion of possible mechanisms for channel formation can be found in ref. [50].

#### F-ATP synthase c-subunit

**Fig. 3:**
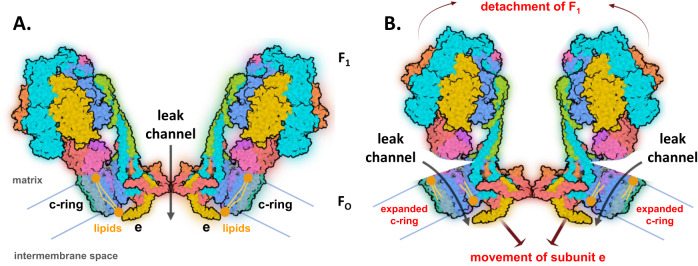
The possible location and conformational changes involved in gating of the mitochondrial F-ATP synthase leak channel. The diagram was drawn according to recent reports [37, 40, 67, 87, 94]. **A** Formation of the pore at the F-ATP synthase monomer interface within the dimer. **B** Reversible opening of the pore within the c-ring under physiological and sub-lethal pathological conditions. Non-reversible dissociation of F_1_ from F_O_ occurs under severe pathological conditions known to induce cell death. Lipids in the c-ring lumen may be displaced or removed due to the expansion of the c-ring allowing ion conduction. F-ATP synthase subunits are shown as surface representations. Gray arrows indicate the possible path of ion flow through the channel. Brown arrows indicate the putative movements of F_1_ subcomplex and e-subunit. Figure created with BioRender.com. PDB ID code: 6ZQN was used to illustrate F-ATP synthase and modified for the depiction of the hypothetical conformational changes in channel opening.

As mentioned earlier, LMNG-purified F-ATP synthase tetramers and dimers but not monomers were reported to form mPT-like channels, suggesting that the pore is located between subunits e and g of each F-ATP synthase monomer [41] (Fig. 3A). In contrast, recent studies have shown that the F-ATP synthase monomer forms a large multi-conductance channel with the biophysical characteristics of mPTP, suggesting that F-ATP synthase monomers form channels, and that dimer formation is not required for channel activity but may also form channels in that a dimer contains two monomers [42, 47] (Fig. 3B).

If the channel resides within the F-ATP synthase monomer, the likely location is the membrane-embedded c-subunit ring, considering its transmembrane, large, pore-like cavity (Fig. 3B). Studies of chloroform-extracted c-subunit from rat liver mitochondria, as well as human c-subunit highly purified by affinity chromatography demonstrate a large conductance, voltage-sensitive channel in patch-clamp recordings [33, 42–47] that has been termed ACLC (ATP synthase c-subunit leak channel). It has also been reported that c-subunit phosphorylation affects channel open probability [46] and that genetic manipulation of c-subunit expression regulates mPTP-driven mitochondrial fragmentation and cell death [44, 47].

The c-subunit ring has been reported to undergo measurable expansion upon mPTP activation by Ca^2+^ in cells [47]. Mutations that loosen the packing of the c-ring increase the internal diameter and channel conductance compared to wild-type c-ring and sensitize neurons [47] and cardiac tissue [86] to death. CypD/Ca^2+^-induced and CsA/ADP-inhibited dissociation of F-ATP synthase F_1_ subunits from F_O_ is associated with mPT, suggesting that unmasking of the c-subunit ring is required for initiation of ACLC conductance [47, 87]. CsA-inhibited dissociation of ATP synthase F_1_ subcomplex from F_O_ has been reported recently in primary hippocampal neurons under glutamate excitotoxicity [87]. Significant reduction in ATP synthase F_1_ subunit levels was observed in glutamate-treated neurons, while the level of c-subunit did not change, suggesting that non-reversible dissociation of F_1_ from F_O_ occurs under severe pathological conditions, which predisposes neurons to cell death [87]. This study also reported that application of purified F_1_ subcomplex consisting of α_3_β_3_γδε subunits inhibits channel activity during patch-clamp recordings of reconstituted c-subunit. Application of α_3_β_3_ complex, which lacks subunits γ, δ, and ε did not inhibit channel activity suggesting that specific interactions between F_1_ subunits and c-ring are required for channel inhibition and that F_1_ constitutes an inactivation gate of ACLC under physiological conditions [87].

The role of ACLC in mPTP has been questioned [79, 88, 89] because of the hydrophobic nature of c-subunit pore-lining residues and the finding that lipids at least partially occupy the c-subunit cavity in structures of F-ATP synthases from bacteria to eukaryotes [90–93]. However, the removal of lipids from the c-ring lumen might be mediated by an interaction of the intracristal space facing lipid with subunit e if combined with appropriate conformational changes as suggested previously for the “Death Finger” model [67, 94]. Nevertheless, removal of the lipids from the c-ring lumen into a hydrophilic environment of the intermembrane space is energetically unfavorable and is unlikely to happen. Instead, lipids could be displaced within the c-ring cavity upon the expansion of the ring that occurs during channel activation (Fig. 3B).

Taking all recent findings together, it may be envisioned that in vivo gating of ACLC requires conformational changes of F-ATP synthase in several phases including 1) the binding of channel activators directly to OSCP subunit [95–97] and/or to β subunit [98, 99] (Fig. 3B); 2) the induction of conformational changes in the F-ATP synthase peripheral stator subunits; 3) movement of central stalk subunits of F_1_ away from the c-subunit on the matrix side and 4) movement of other F_O_ subunits, including expansion of the c-ring. It is possible that lipids are displaced within the c-ring cavity or removed to open ACLC during these events as suggested recently [42, 50, 67, 84, 94] (Fig. 3B), although there is currently no high-resolution structure of the open channel conformation of the ACLC to support this proposal. The model illustrates reversible brief openings of ACLC that occur under physiological and sub-lethal pathological conditions. In addition, non-reversible dissociation of F_1_ from F_O_ may occur under severe pathological conditions and initiate mitochondrial swelling and cell death [84, 87].

#### ANT

Over thirty years ago, the pore-forming component of the mPT was hypothesized to be the ANT. This was due to several independent observations that ANT inhibitors and substrates greatly affect mitochondrial Ca^2+^ retention capacity (CRC) and mPTP sensitivity [35, 100–102]. In addition, the ANT directly interacts with CypD, the well-established sensitizer for the mPTP, and reconstitution experiments revealed that the ANT is able to form high conductance channels [80, 103–106]. Furthermore, oxidative stress and its mimic, phenylarsine oxide, that are potent sensitizers of pore opening, specifically cross-link two cysteines on the ANT and greatly reduce the ability of ADP to inhibit pore opening [35]. Interestingly, however, genetic deletion of both of the predominant isoforms of the ANTs (*Ant1* and *Ant2*) in the mouse liver revealed that these mitochondria were still sensitive to Ca^2+^-induced mitochondrial swelling and responsive to CypD inhibition by treatment with CsA [28]. These ANT1 and ANT2-null mitochondria did, however, have significantly increased mitochondrial CRC and a desensitized mPTP compared to wild type control mitochondria [28]. In fact, mitochondria lacking CypD or treated with CsA have similar levels of desensitization to the ANT1 and ANT2-null mitochondria, and CsA treatment further desensitizes the mPTP in the ANT1 and ANT2-null mitochondria [28, 107, 108]. Taken together, these data suggest that the ANTs and CypD both contribute to mPTP opening and may act independently of one another.

The role of the ANTs as the mPTP was reexamined recently by genetically removing all murine isoforms of the ANT family (*Ant1*, *Ant2*, and *Ant4*) in the mouse liver to rule out any compensation by ANT4. The results confirmed that mitochondria lacking the ANTs are highly resistant to Ca^2+^-induced mPTP opening. However, in response to extremely high levels of Ca^2+^, the mPTP does engage and mitochondria undergo swelling [32]. Surprisingly, treatment of these mitochondria with CsA completely inhibits mPTP opening, as confirmed by the absence of mitochondrial swelling, while Ca^2+^ uptake reaches maximum capacity [32]. There are two potential explanations for these data. The first is that CypD or the ANTs independently regulate a common pore-forming component of the mPTP. The second is that the mPTP is comprised of two distinct pores, one formed by the ANT family, which may or may not be influenced by CypD, and another pore, which requires CypD to engage and may involve the F-ATP synthase as described earlier. However, the controversy remains due to the studies from the Walker laboratory reporting that genetic removal of critical components of the F-ATP synthase does not appear to affect mitochondrial CRC or Ca^2+^/CypD-dependent swelling [88, 89, 109], which could be due to the remaining contribution of the ANT family. In support of this, channel activity sensitive to the known ANT inhibitor, bongkrekic acid (BA), is present in mitoplasts isolated from c-subunit lacking cells [33]. Mitoplasts isolated from cells lacking subunit c or subunit g have currents that are of smaller conductance and more difficult to elicit with Ca^2+^[81, 87], but still manifest BA-sensitive, ANT-like currents.

There remain several unanswered questions regarding the relationship between the ANT family and the mPTP. For instance, which pore-forming component of the mPTP is the most relevant to pathologies involving necrotic cell death? Deletion or inhibition of CypD is protective against a wide variety of these diseases [107, 108, 110–112], suggesting that the CypD-dependent pore may be the most advantageous for therapeutic targeting. It should be noted, however, that while the ANT-pore may not require CypD to engage, CypD might sensitize the opening of this channel. Thus, determining if, and how, CypD alters the ANT-pore remains an important question to be addressed. Finally, and possibly most importantly, what is the molecular identity of the CypD-dependent pore that leads to permeability transition? This is critical knowledge and should be interrogated in the absence of the ANTs such as in [47] or following their inhibition. Overall, it is evident that the ANT family does play a critical role in mPTP formation. However, the existence of additional CypD-dependent pore-forming components such as the F-ATP synthase has likely obscured the contribution of the ANT family. Addressing these lingering questions is critical to the goal of identifying novel therapeutic approaches that lead to the complete inhibition of the mPTP, which could be beneficial for numerous diseases involving necrotic cell death.

## Enigmatic dual roles of F-ATP synthase and ANT in both ATP generation and formation of the mPT pore

Identification of specific proteins (e.g., F-ATP synthase and ANT) responsible for mPTP formation in addition to their normal physiological roles provides the opportunity to generate detailed models at the atomic level of how large pores open within these usually tightly coupled structures. It is the general assumption that mPTP is a Ca^2+^-activated, large conductance, non-selective ion channel. For mPTP to fit into this “generic” model, it would be expected that the candidate channel forming protein or its regulatory proteins would have a specific site that can bind Ca^2+^ and induce the conformational changes that cause channel opening [36, 83]. In agreement with this prediction, patch-clamp assays show that the addition of Ca^2+^ activates mPTP-like channels in the inner membrane associated with both F-ATP synthase and ANT [33, 47, 113]. Similarly, purified ANT and F-ATP synthase proteins reconstituted into model lipid membranes form large conductance pores following the addition of high concentrations of Ca^2+^ [41, 42, 80]. This suggests that it might be possible to use published structures of the intact ANT and F-ATP synthase complexes to explain how mPTP activation occurs. However, there are two key features of the properties of the mPTP in intact mitochondria that are different from those of the purified channel proteins. Firstly, concentrations of Ca^2+^ required to activate mPTP in patch-clamp experiments are two to three orders of magnitude higher than the concentrations of free Ca^2+^ that induce mPTP in intact mitochondria [114]. Secondly, a single mitochondrion has fewer than ten mPTP channels activated during the mPT [48, 49, 115]. Considering that each organelle has several thousand copies of the ANT and F-ATP synthase in the native conformations, this suggests either that the probability of these proteins taking on an open channel conformation is extremely low, or that there may be other, as yet unidentified, proteins expressed at low frequency that comprise the mPTP [48, 49]. If the former is the case, it will be important to uncover the mechanisms underlying how such a small subpopulation of the ANT and F-ATP synthase proteins can be responsible for mPTP formation. This may act as a limitation when trying to use the known “normal” structures of the ANT and F-ATP as a foundation for finding the structure of the active channel of the mPTP.

The discrepancy between the Ca^2+^ concentrations required for mPTP activation in intact mitochondria and those required by reconstituted channel proteins could also be explained if Ca^2+^ activation in intact mitochondria does not occur directly through the putative ANT or F-ATP synthase binding site but at a distal location within the mPTP regulatory protein complex. In this case, the channel activation mechanism would require Ca^2+^-induced structural changes. Indeed, studies indicate that during Ca^2+^ uptake, free mitochondrial Ca^2+^ reaches a plateau and becomes efficiently buffered by phosphate species long before mPT is activated [114, 116]. This may indicate that in intact mitochondria, mPT is not activated directly by free Ca^2+^ binding but through more complicated interactions, perhaps involving the formation of complexes between Ca^2+^ and other molecules [117]. One such molecule is cardiolipin, the binding of which to both the ANT and the F-ATP synthase is essential for their activity. Interestingly, unlike most Ca^2+^-binding proteins, cardiolipin binds Ca^2+^ differently from Sr^2+^ perhaps explaining why the latter does not activate but rather inhibits the mPT [118]. Another mechanism might involve the hypothetical existence of Ca^2+^ and/or Ca^2+^-mediated ROS “hot-spots” inside mitochondria where local Ca^2+^ reaches concentrations that are much higher than average concentrations measured experimentally. In this respect, it will be interesting to investigate the association between the mPTP and the high capacity Ca^2+^ uptake channel mitochondrial Ca^2+^ uniporter (MCU). If the mPTP and MCU protein complexes are in close proximity, Ca^2+^ influx through the MCU could create microdomains of elevated Ca^2+^ concentrations sufficient to induce mPTP formation. Interestingly, in adult cardiomyocytes, MCU is discretely localized in the IMM that is in proximity to the junctional sarcoplasmic reticulum, which releases Ca^2+^ via ryanodine receptors during excitation-contraction coupling cycles [119].

The presence of a very small number of mPTP channels in intact mitochondria might also be explained by the specific morphological features of the mitochondrion. It is possible that in intact mitochondria, formation of the mPTP requires the presence of not only the core components (either ANT or F-ATP synthase) but also of other proteins, most notably VDAC—an outer membrane channel that is believed to be associated with the mPTP complex. It should also be noted that mPTP is a voltage sensitive channel. As such, it is conceivable that its open probability might be quite low at the polarized mitochondrial membrane potential. It has been proposed that such a “supercomplex” could be present preferentially at contact sites between the outer and inner membrane [23, 120, 121]. The importance of interactions between various components of mPTP complex is further supported by a recent finding that in intact cells mPTP is not activated if either ANT or assembled ATP synthase is not present [122]. Interestingly, these cells still undergo Ca^2+^ induced, CsA dependent depolarization but lack high-conductance permeability. Furthermore, mPTP may have the highest probability of being activated only at these specific sites. As a result, the number of pores is orders of magnitude smaller than the number of ANT and F-ATP synthase copies. Alternatively, if mPTP activation requires close proximity to the MCU, this will decrease the number of pores dramatically due to a smaller number of functional MCU complexes and their discrete distribution. Another possibility, supported by inhibitor binding studies [48] is that only a small subpopulation of the proteins enter specific conformations that are different from their native conformation, and that would allow them to transform into mPTP. For example, mPTP could be activated only in complexes that are not fully assembled or that are partially misfolded, perhaps due to oxidative stress that may alter protein conformations. If this is the case, the knowledge of the atomic structures of native proteins might not be sufficient to completely resolve the mPTP structure.

Notably, all current models are based on the structures of the intact proteins found in the mitochondria under normal conditions. One of the exciting perspectives will be to obtain high-resolution structures of these proteins under stress conditions. A first attempt to work in this direction is the recent cryo-EM structure of the F-ATP synthase in the presence of very high (5 mM) Ca^2+^ [67]. Importantly, one of the key features seen in these cryo-EM images is the possible displacement of the F_1_ ATP synthase during mPTP activation, which confirms functional data that demonstrate inhibition of the F_O_ channel by purified F_1_ complex [87]. Interestingly F-ATP synthase under these conditions generates multiple structures including native, unaltered structures and highly distorted protein structures. However, this study did not demonstrate well-defined pores in these distorted structures which suggests that even at very high concentrations of Ca^2+^, there is no clear transformation pathway of the native F-ATP synthase into a large mPTP channel. This is also consistent with the presence of mPTP-like channels of various conductances observed in patch-clamp experiments ranging from large pores (reviewed in ref. [123]) to small conductance leak [124].

It is also worth noting that recent studies demonstrated that the c-subunit of the ATP synthase is an amyloidogenic peptide capable of forming oligomeric β-barrel large pores and this process is CypD dependent [125, 126]. It is tantalizing to hypothesize that stress conditions might lead to the formation of misfolded c-subunits, which would induce the mitochondrial toxicity pathway similar to mechanisms established in experiments with other known pore-forming amyloid proteins [127].

## The roles of mPTP in health and disease

The recognition that the candidate proteins responsible for mPTP are also responsible for ATP generation suggests that these proteins are walking a fine line between cell life and death. Here we will briefly highlight a few key paradigms in physiological and pathological settings that appear in the current literature.

### The physiological roles of mPTP

The physiological roles of the mPTP have been appreciated recently [14, 24, 86, 128]. The general consensus is that transient opening of the mPTP (t-mPT) can regulate mitochondrial Ca^2+^ efflux [129, 130], ROS signaling [131, 132], life span [133, 134], cellular metabolism [135] and differentiation of neuronal, cardiac muscle and stem cells [136–138]. It is well recognized that frequency modulation rather than amplitude modulation comprises an important physiological mechanism for encoding cellular signals. Therefore, oscillatory brief openings of the mPTP would be appropriate for carrying out several physiological functions as mentioned above. However, what mechanisms govern the stochastic nature of t-mPTP in the domain of space and time is still a mystery. For instance, in adult murine cardiomycytes, the t-mPTP mediated mitoflash occurs approximately four times/1000 μm^2^/100 s [131]. Therefore, t-mPTP could generate certain localized molecules that initiate a signal cascade leading to a longer and broader cell regulation such as post translational modification of a specific set of proteins. These unique characteristics in the physiological role of mPTP are elaborated below.

#### MPTP as a Ca^2+^ release channel and beyond

In respiring mitochondria, Ca^2+^ influx is primarily mediated by the MCU while Ca^2+^ efflux is largely carried out by Na^+^/Ca^2+^/Li^+^ exchange (NCLX). Collectively, through sophisticated coordination, these pathways maintain steady state mitochondrial free Ca^2+^ concentrations ([Ca^2+^]_m_) in the physiological range of 0.1–1 µM. This is despite the Δ*Ψ*_m_ being about −180 mV, which is a huge driving force that unopposed would cause massive accumulation of Ca^2+^ within the matrix. The rate of Ca^2+^ uptake increases very steeply with extramitochondrial [Ca^2+^], reaching a Vmax as high as 1400 nmol Ca^2+^ × mg protein^−1^ x min^−1^; while the maximal rate of operation of the antiporter is not faster than about 20 nmol Ca^2+^ × mg protein^−1^ × min^−1^. This difference could expose mitochondria to the risk of Ca^2+^ overload. However, under physiological conditions, mitochondrial Ca^2+^ uptake is relatively small during the rapid oscillatory Ca^2+^ transients that occur, for example, in beating heart. In non-excitable cells or when frequencies of Ca^2+^ transients through GPCR mediated Ca^2+^ release from IP_3_ receptors are low, NCLX along with high matrix Ca^2+^ buffering capacities can maintain cytosolic and mitochondrial Ca^2+^ homeostasis globally. Meanwhile, the existence of stochastic, infrequent, and localized transient mitochondrial membrane depolarization with matching transient mitochondrial Ca^2+^ release due to flickering of mPTP under physiological conditions has been widely recognized [139–141]. In neurons these events occur constantly during normal physiological activities such as accompanying action potential-induced plasma membrane depolarization and during synaptic transmission. Therefore neuronal mPT is likely to be well-adapted for Ca^2+^ release [142, 143]. These localized events appear to provide a convenient mechanism for t-mPT to serve as a safety valve to rapidly discharge excessive Ca^2+^ accumulation in a small subset of mitochondria [130]. An interesting question is why nature would design a non-selective ion channel for mitochondrial Ca^2+^ release. The answer is probably that the very low permeability of the inner membrane, which is a requisite for energy conservation, would prevent rapid Ca^2+^ release even after a full depolarization of Δ*Ψ*_m_ [130]. Furthermore, since the mPTP is a multi-conductance channel, the low frequency of t-mPT may be associated with a low-conductance mode and thus might allow for maximal Ca^2+^ flux without the need for persistent membrane depolarization. This arrangement in turn permits fast Ca^2+^ release even for very small [Ca^2+^] gradients, and is consistent with the protective effect of pore flickering in several disease paradigms [144–146].

Another important role for the “physiological mPTP” may be to alter cell metabolism in favor of aerobic glycolysis over oxidative phosphorylation. The metabolic shift away from aerobic glycolysis occurs in a time-dependent manner at various key points in development [147]. One example is in neuronal synapse development, where the ATP synthase c-subunit leak channel (ACLC) becomes relatively more closed in a synaptic stimulus-dependent manner during synapse maturation. The mechanism is related to rapid movement of the anti-apoptotic protein Bcl-xL to mitochondria [148] followed by assembly of newly synthesized F-ATP synthase F_1_ components with free c-subunit rings, the latter of which are already present in the mitochondrial inner membrane [128]. This new synthesis adds newly assembled ATP synthase to that already existing. If this increased ATP synthase assembly fails to occur at a critical time point, synapses do not develop normally. The function of the free c-subunits earlier in development may be to provide a persistent inner membrane leak [149] that supports an aerobic glycolytic metabolic phenotype [128]. Aerobic glycolytic metabolism allows for a high rate of protein synthesis [128] and perhaps lipid biosynthesis during the developmental period. The study suggests that the high rate of protein synthesis associated with the glycolytic metabolism normally decreases at the time of synapse maturation, regulated by relative ACLC closure.

As mentioned earlier, Ca^2+^ release is only one of numerous signals that may be associated with t-mPT. These include transient bursting of ROS and ATP generation, alkalinization of matrix pH, and occurrence of fluxes of other ions like Na^+^. These t-mPT mediated localized signals could have profound effects on cell regulation including development, differentiation, maturation and aging, and localized organelle-organelle crosstalk signaling. The t-mPTP openings in intact cells have been detected through small molecule or protein-based fluorescence probes and mitochondrial ion channel recordings. Although living cell ion channel recordings are ideal to detect rapid response kinetics, they fail when attempting to visualize pan-cellular mitochondrial events, and imaging techniques are not yet sensitive enough to record rapid and smaller mPTP flickers. Future development of new probes coupled with advanced imaging techniques may be able to address the question of whether t-mPT occurs only in a very small subset of mitochondria or if it is a more universal event for the mitochondria.

### The pathological roles of the mPTP

#### Contribution of the mPTP to necrotic and apoptotic death pathways

The mPTP plays key roles in cell death pathways, especially in necrotic cell death, but its role in canonical apoptosis is still controversial. Classically Bcl-2 mediated death does not require CypD dependent mPTP activation, although disruption of inner membrane activities including impairment of electron transport, occurs during apoptotic events. Rita Levi-Montalcini and colleagues were among the first to recognize programmed cell death in neurons in developing chick embryos. She discovered that nerve growth factor was required to prevent death of sympathetic and sensory neurons in the peripheral nervous system [150]. Later work by many groups led to the discovery of apoptosis [151], and the recognition that growth-factor withdrawal dependent cell death was related to proapoptotic Bcl-2 family proteins such as Bax [152–154]. These proteins may also modulate mPTP formation through interactions at contact sites between the inner and outer mitochondrial membrane and changes in mitochondrial morphology [155]. While mPTP opening itself may play a role in apoptosis as discussed further below, there exist many other recognized forms of cell death, in addition to canonical apoptosis and necrotic death [156]. Since the role of the mPTP in several additional cell death mechanisms is unclear, they will not all be discussed further here.

Apoptosis is characterized by cell shrinkage, membrane blebbing, condensation of the chromatin (pyknosis) [151], and activation of caspase proteases [157, 158]. Intrinsic apoptotic cell death is caused by stress experienced autonomously by a cell such as mediated by oncogenes, direct DNA damage, hypoxia, and survival factor deprivation. [159–162].

Mitochondria regulate caspase dependent cell death through pro-apoptotic Bcl-2 family members such as Bax/Bak which mediate mitochondrial outer membrane permeabilization (MOMP), leading to formation of the apoptosome and release of cytochrome c which activates the caspase-cascade [163]. MOMP is preventable by high levels of anti-apoptotic proteins such as Bcl-xL [164, 165] and the balance between pro-and anti-apoptotic members of the family contributes to the commitment point on the road to apoptotic cell death [166]. However, it has also been shown that in intact cells cytochrome c release may also occur after acute or prolonged increases in intracellular Ca^2+^ and mitochondrial Ca^2+^ overload in a Bax/Bak independent manner [167]. Swelling of mitochondria in response to mPTP opening may lead to cytochrome c release by outer membrane rupture, but also by increasing cytochrome c availability through the Bax/Bak pathway after cristae remodeling [168]. This may stimulate apoptosis in the presence of preserved ATP levels. In severely damaged cells necrotic cell death is unavoidable and is characterized by cell swelling, plasma membrane rupture, and loss of organellar structure without chromatin condensation [158, 169] . This is exemplified by ischemia reperfusion injury where a key role for mPTP opening has been most firmly established [158, 169] and is discussed further below.

Although necrotic death is not genetically regulated during severe injury, in many cells that survive the initial insult, a programmed set of pathways is activated that is quite different from apoptotic death. This death, called necroptosis, is highly regulated, caspase independent, and resembles only morphologically some aspects of necrosis. The main molecular player for necroptosis is protein RIP kinase 3 and its substrate the Mixed Lineage Kinase Domain-like Protein (MLKL), the latter of which forms the executioner by permeabilizing the plasma membrane. Necroptosis is usually initiated extrinsically to the cell by high levels of death signals in the extracellular space, such as TNF, which are typically found in certain severe acute, degenerative or inflammatory states. The complex necroptotic pathway, including activation of RIP kinases, has many decision-making forks downstream of TNF receptor activation that can lead, variously, to survival, apoptosis (which is immune silent) or activation of cell lysis and pro-inflammatory programs. Although the link between apoptotic signaling and mPTP activation may at times be direct as outlined in the preceding section, the mechanism of activation of mPTP during necroptotic cell death is not yet clear, but a convincing case has been made for ischemia- and oxidative stress-induced myocardial necroptosis via CaMKII [170]. It should be noted that this CamKII mediated cell death is independent of MLKL, thus may not be defined as necroptosis according to consensus in the cell death field [167]. The MLKL protein is an executioner of necroptosis and its N-terminal helix bundle can bind the mitochondria-specific phospholipid, cardiolipin [171, 172]. This effect causes permeabilization of the mitochondrial inner membrane resembling mPT, accompanied by cell and organelle swelling. Indeed, cells lacking CypD and therefore resistant to mPT are resistant to necroptotic cell death induced by TNFα and caspase inhibition [173]. However, in genetic model systems, It was shown that CypD is dispensable for necroptosis [174]. Moreover, other studies showed that activated MLKL translocates uniquely to the plasma membrane, where it interacts with PIP phospholipids during the process of cell death [175, 176]. MLKL lacks a mitochondrial targeting sequence, and forcibly targeting MLKL to mitochondria with the use of selective tags does not induce cell death [177]. Necroptotic signaling factors can also interact with metabolic enzymes, hampering ATP production [178], cause loss of NAD through DNA damage pathways, activate ROS production and precipitate loss of mitochondrial and plasma membrane potential, all of which may activate mPT. The late events in necroptosis also involve rapid activation of calpains, Bax and release of pro-apoptotic factors into the cytosol [179]. However, unlike apoptosis, mitochondrial depletion does not compromise the ability of a cell to undergo necroptosis, suggesting that mitochondria play a regulatory role in this cell death pathway [174].

#### The role of mPTP in innate immunity

Another important role for inner membrane permeabilization by mPTP is related to mitochondrial DNA release during innate immunity. Certain extracellular signals known as pathogen or danger associated molecular patterns interact with toll-like receptors (TLRs) on macrophages [180–182] . Signaling downstream of TLRs helps to inactivate extracellular pathogens by formation of an intracellular complex called the inflammasome [183]. The inflammasome is often further activated by newly synthesized mitochondrial DNA [184–186], some of which is exposed to oxidative stress. Oxidized mitochondrial DNA is released through mPTP into the cytosol [183, 184] and can further propagate the inflammatory signal to neighboring cells through mitochondrial DNA exocytosis [187, 188]. However, mitochondrial DNA release may not be entirely dependent on mPTP since other studies have shown that activation of Bax/Bak can lead to mitochondrial herniation and DNA release [189, 190]. Notably, this method of mitochonrial DNA release was indepdent of CypD as cells lacking CypD still underwent Bax/Bak-dependent mitochondrial DNA release [190]. Given these data, it is clear there is a relationship between MOMP, mPTP sensitization, and inner membrane integrity that remains to be explored [191, 192].

#### The role of the mPTP in ischemia/reperfusion injury

Reperfusion of the heart (and other tissues) following an extended period of ischemia can exacerbate the damage caused by ischemia itself and is known as ischemia reperfusion injury (IRI) [118, 193]. It has long been known that ischemia is associated with elevated intracellular [Ca^2+^] and phosphate and that reperfusion causes the production of ROS. These are exactly the conditions that would be expected to cause mPTP opening and this was subsequently shown to be the case in both isolated heart cells [194] and the perfused heart [187]. Furthermore both pharmacological [188] and genetic [107] attenuation of CypD activity was shown to protect from IRI, and it is now widely accepted that opening of the mPTP during reperfusion plays a key role in IRI in a range of tissues. Indeed, inhibition of mPTP opening can be induced by a variety of pharmacological, preconditioning and post-conditioning protocols that lead to cardioprotection in models of IRI [10, 118, 193, 195]. However, CsA itself has not proved to be an effective cardioprotective agent in some animal models of IRI or in large scale clinical trials [193]. This probably reflects the ability of the mPTP to open independently of CypD if the trigger is sufficiently large [118, 196]. Furthermore, if F_1_ is lost during prolonged cellular toxicity, as was shown recently, CsA and other reagents that bind within the F_1_ might no longer inhibit the channel [87]. There exist areas of uncertainty in our understanding of how the mPTP mediates IRI. First, what is the primary trigger of mPTP opening in reperfusion; is it increased [Ca^2+^], ROS or some other factor? Second, how do cardioprotective strategies such as ischemic preconditioning (IP) inhibit mPTP opening?

It seems highly probable that the observed rise in intracellular [Ca^2+^] during ischemia plays a key role in priming mitochondria for mPTP opening during reperfusion when re-energisation leads to uptake of the accumulated Ca^2+^ into the mitochondria [197]. In support of this view, pharmacological or genetic inhibition of mitochondrial uniporter activity does reduce IRI [197–199] as does over expression of the Na^+^/Ca^2+^ exchanger that mediates Ca^2+^ efflux from the mitochondria [200]. The other widely accepted trigger for mPTP opening during reperfusion is increased ROS production. This has long been recognized to accompany reperfusion, although targeting ROS as a cardioprotective strategy has proved disappointing [10, 195]. Recently, Murphy and colleagues have presented evidence that succinate, which accumulates greatly during ischemia, is rapidly oxidized by reverse electron flow leading to superoxide production at Complex I [201]. However, using surface fluorescence of the Langendorff perfused heart others found no evidence for increased ROS production in the first 2 minutes of reperfusion, when mPTP opening is triggered. Rather, ROS production occurred later in reperfusion as a consequence of mPTP opening and was largely prevented by inhibiting mPTP opening with CsA or IP [202]. Furthermore, measurements of mitochondrial NADH and flavoprotein fluorescence show that a highly reduced state of Complex I and energisation of mitochondria are not maintained in the early phase of reperfusion, yet this is required for ROS production during reverse electron flow at Complex I [202, 203]. In addition, the rapid decrease in tissue succinate levels during the first few minutes of reperfusion can largely be explained by succinate efflux from the heart on the monocarboxylate transporter MCT1 [203, 204]. However, it remains possible that mitochondrial ROS is a trigger for the initial mPTP opening, but that the levels are below the limit of detection by the ROS probes used. Nevertheless, studies on the mechanism of IP suggest that other factors are more important in sensitizing the mPTP to [Ca^2+^] and causing its opening in the first minutes of reperfusion as outlined below.

**Fig. 4:**
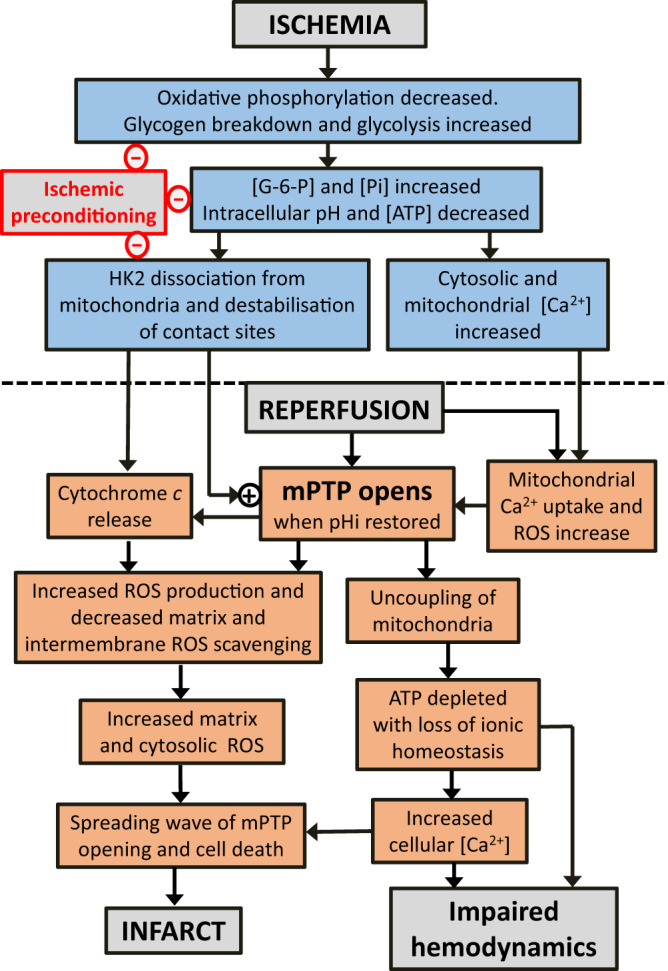
Possible mechanisms involved in the opening of the mPTP in ischemia reperfusion injury and its prevention by ischemic preconditioning. This diagram summarizes key factors that have been proposed to initiate and amplify mPTP opening during reperfusion of the heart after a period of prolonged ischemia which itself does not cause mPTP opening because of the low ischemic pHi. Events occurring primarily during ischemia are indicated in pastel blue and those during reperfusion in salmon. The sites at which ischemic preconditioning (IP) may attenuate mPTP opening and hence reduce reperfusion injury (infarct) are indicated with a red minus sign. Further details are given in the text and [123, 182, 190]. This scheme is a modified version of that presented previously [203].

IP involves exposing the heart or other organs to brief periods of ischemia, with intervening reperfusion, prior to prolonged ischemia [119, 205]. It leads to potent ischemic protection that is associated with inhibition of initial mPTP opening and enhanced subsequent mPTP closure during reperfusion [206]. However, the mechanisms responsible and signaling pathways involved remain a matter of considerable debate [10, 119, 193]. They cannot be explained simply by decreased mitochondrial Ca^2+^ accumulation during reperfusion or by lower ROS production during early reperfusion [202]. Protein kinases including PKCe, PKA and reperfusion injury signaling kinase have all been implicated [119, 193]. However, there is no consensus as to how these might regulate the mPTP. Mitochondria isolated from preconditioned hearts after ischemia exhibit a marked decrease in sensitivity to Ca^2+^ induced mPTP opening when compared to ischemic mitochondria from non-preconditioned hearts, but no significant changes in the phosphorylation state of any of the proposed components of the mPTP were observed that correlated with cardioprotection [207]. More recently it was shown that CypD may be regulated by phosphorylation or a range of other modifications [208], but data correlating the extent of these modifications with cardioprotection is lacking. By contrast, a strong correlation has been observed between the extents of hexokinase 2 (HK2) dissociation from mitochondria at the end of ischemia with the extent of mPTP opening on reperfusion [209]. HK2 is usually tightly bound to the outer membrane of heart mitochondria through a short conserved hydrophobic α‐helical domain in the N‐terminus, but the decrease in pH and increase in glucose-6-phosphate that occur in ischemia can cause its dissociation and both these parameters are attenuated by IP [155, 210]. The mechanism by which bound HK2 protects mitochondria from mPTP opening remains uncertain but may involve decreased binding of the fission factor Drp1 to the outer membrane during ischemia [211] and preventing Ca^2+^ discharge into mitochondria from mitochondria-associated membranes of the endoplasmic reticulum [212]. Indeed pharmacological and genetic attenuation of Drp1 binding to mitochondria is cardioprotective and several known phosphorylation sites on Drp1 may provide additional regulatory mechanisms linking the protein kinase signaling pathways in preconditioning to mPTP inhibition [155, 213]. It has been proposed that the effects of Drp1 on mitochondrial cristae morphology disrupt contact sites between the inner and outer mitochondrial membranes leading to sensitization of the mPTP to [Ca^2+^] [209, 211]. Prevention of HK2 dissociation and thus Drp1 binding by IP could explain the observed attenuation of mPTP opening early in reperfusion. This, in turn, will prevent subsequent rises in ROS and [Ca^2+^] that, in unprotected hearts, will cause an increasing cascade of further mPTP opening, ROS production and [Ca^2+^] dysregulation leading to necrotic cell death (infarct). This is illustrated schematically in Fig. 4.

Interestingly, at the periphery of the infarct there may be an area of complex cell death and survival as cells deal with Ca^2+^ overload, oxidative and ER stress and inflammation [214]. This might reflect mPTP opening that is sufficient to cause matrix swelling, outer membrane rupture and cytochrome c release from some mitochondria that initiates the apoptotic cascade but without the overall bioenergetic compromise that leads to necrosis [215].

In ischemic cell Ca^2+^ overload, the mitochondrial inner membrane is the first location of death channel (mPTP) activation before the outer membrane Bcl-2 machinery [216, 217] which activates mPTP. The dysregulated Ca^2+^ accumulation that occurs in the necrotic core leads to opening of mPTP [218], but mPTP can also be activated in the surround of the core or penumbra. mPTP opening leads to mitochondrial depolarization, inhibition of ATP synthesis and finally Bax activation, as the outer membrane gets involved. This then results in the release of pro-death factors into the cytosol including cytochrome c [219–221]. During ischemic or toxic cell death, anti-apoptotic proteins such as Bcl-2 and Bcl-xL can be proteolytically cleaved by caspases and calpain to form pro-death molecules. The N-terminal cleaved version of Bcl-xL has been implicated in neuronal ischemic cell death and in activation of Bax [222]. The effect of cleaved Bcl-xL on inner mitochondrial membrane depolarization is inhibited by Bcl-xL inhibitors and by CsA, suggesting its direct action on mPTP. ATP synthase c-subunit knock down also protects against mitochondrial inner membrane depolarization during glutamate toxicity, suggesting that the mechanism involves initial activation of an mPTP-associated channel [24, 44].

#### The role of ATP synthase c-subunit leak channel (ACLC) in neurodegenerative disease

ACLC has recently been shown to play a role in human health and disease and has therefore become an important target to prevent brain and cardiac ischemia and neurodegeneration. Decreases in F_1_ content compared to F_O_ have been reported in rat brain and heart mitochondria during aging [223, 224]. The α subunit of the mitochondrial F-ATP synthase is the most common lipoxidation protein in human Alzheimer Disease (AD) brains at early Braak stages [225], leading to stoichiometric changes in F-ATP synthase in degenerating neurons [226]. In the brains of AD individuals and in an AD mouse model, a decrease in F-ATP synthase OSCP subunit level [227] is related to F_1_ dissociation from F_O_ in neuronal mitochondria [227]. Similarly, loss of the F-ATP synthase F_1_ β subunit and reduction in F_1_/F_O_ ratio occur in a DJ-1 deficient, Parkinson’s disease (PD) mouse model [228, 229]. DJ-1 binds directly to the F-ATP synthase β subunit and this interaction decreases mitochondrial uncoupling and enhances ATP production efficiency [228]. In contrast, DJ-1 deficient mitochondria demonstrate low F-ATP synthase β subunit levels, reduction in F_1_/F_O_ ratio and increased open probability of ACLC. These changes correlate with decreased ATP synthesis efficiency [228, 230], low cytosolic ATP levels and reduced neuronal growth.

#### The role of the mPTP in muscle diseases

The hypothesis that Ca^2+^-dependent mitochondrial dysfunction plays a role in muscle diseases was suggested 47 years ago [231], and thereafter supported by studies in a variety of disease models (reviewed in [232]). Important developments have been the demonstration that the mPTP plays a pathogenic role in muscular dystrophy caused by collagen VI [233], dystrophin, δ-sarcoglycan and laminin deficiency [234] as well as in muscle atrophy of aging [235] and denervation [236]. The involvement of the mPTP in pathogenesis has provided novel pharmacological strategies based on pore inhibition [237].

#### The role of mPTP in aging

Triggers for the mPT in cells in culture in vitro or ex vivo, which include oxidative stress, energy depletion, and high cytoplasmic Ca^2+^ concentrations [2, 3, 238] are well described. However, these triggers are largely unachievable in vivo except in extreme circumstances such as ischemia or reperfusion injury [239]. Thus, major contributors to the mPT in vivo in aging remain less clear and have been extrapolated by logical extension, for example, by common inhibition of both the decline in mitochondrial membrane potential in aging tissues and of the mPT in cells in vitro by the CypD inhibitor CsA [240].

A growing series of compelling data indicate that the mPTP has a greater propensity to open in aging [241]. Firstly, mitochondrial membrane potential and efficiency of oxidative phosphorylation decline with age, across multiple tissues and in many model organisms [240, 242–245]. While this could implicate factors such as accumulated damage to the respiratory chain, evidence indicates that increased mPT may play a causal role in the decline in mitochondrial membrane potential and decline in ATP generation with age [240, 246]. Secondly, a major stimulus for the mPT, increases in oxidative stress and associated damage of cellular structures, is a central hallmark of the aging process [247]. Evidence in model organisms indicates that primary exposure to mitochondrial ROS may prompt a hormetic response, priming stress-defense pathways to protect against further oxidative insults [248, 249]. As organisms age, however, oxidative insults prompt cellular damage that declining defense mechanisms cannot ward off, driving mitochondrial and organismal decline [250]. Thirdly, and perhaps most compellingly, indirect measurements of mPTP opening in isolated mitochondria or permeabilized cells indicate that a signature common to multiple tissues in aging is enhanced mPTP activation [240, 251–253].

It should be stressed that these measurements were made by exposing cells or mitochondria to non-physiologic stimuli known to induce the mPT such as high concentrations of Ca^2+^ and inorganic phosphate [238, 254]. Less perturbational methods for assessing mPT in aging require exposing cells or mitochondria to Ca^2+^ sensitive fluorophores, non-endogenous metabolites, or examination of structural changes in mitochondria such as swelling [187, 255–257]. However, even the latter has been examined to a greater extent upon stimulation with non-physiologic levels of Ca^2+^ [258]. It should also be noted that changes in mitochondrial architecture and swelling are likely to have multiple causal factors during aging, with mPT representing but one. Finally, highly sophisticated microscopic techniques using genetically encoded sensors now permit the direct, real-time visualization of bursts of mitochondrial superoxide, so called “mitoflashes” [131, 140]. Mitoflash frequency is highly correlated with the rate of aging and organismal decline in *C. elegans* [133]. However, in spite of the idea that mitoflash is strongly correlated with mPTP opening [259], imaging techniques demonstrate changes suggestive of increased mPTP activity rather than provide a direct measurement of mPT, and the relevance of mitoflash in aging has been called into question [260, 261].

Cellular consequences of the mPT differ depending upon the strength and context in which mPTP opening occurs. As noted above, short-term mPTP opening may play a role in mitochondrial Ca^2+^ or metabolite homeostasis and more complex cellular events, for example in facilitating cellular pluripotency [16, 262–264]. Non-sustained mPTP opening has been further shown to play a role in mitochondrial quality control and degradation by autophagy [265–271]. Alternatively, sustained, widespread mPTP opening leads to ATP depletion, induction of autophagy, and necrotic or apoptotic cell death [271–273]. While it is clear that opening of the mPTP is linked to cellular apoptotic and necrotic cell death, whether it is the summation of cellular-level effects on cell death or some other behavior of the mPTP that contributes to aging-related decline and ultimately organismal death remains unknown. In this case we draw sharp distinction between obvious acute insults that trigger mPT such as ischemia-reperfusion injury [107, 274] versus stimuli that serve to prompt mPT across the lifespan.

Recent work from the Soukas and Lithgow laboratories suggest that genetic stimulation of mPTP opening is sufficient to shorten lifespan in an mPTP-, autophagy- and mitochondrial unfolded protein response-dependent manner [11, 134]. These data permit several important conclusions to be drawn. Firstly, although the role of VDAC1 in mPT in cells in culture has been called into question in loss of function experiments [29], in support of prior data, enhanced expression of VDAC1 protein (either by overexpression or by loss of function of the kinase Sgk1 that leads to an accumulation of VDAC1 protein) both facilitate mPTP opening in cells in culture in vitro and shorten lifespan in *C. elegans* in vivo [11]. Secondly, and importantly, the inhibition of autophagy alone when mPTP opening is stimulated is sufficient to reverse shortened lifespan, indicating that it is not enhanced mPT alone that executes the detrimental effects of mPTP opening on aging, but rather the cellular context in which mPT occurs. Thirdly, genetically triggered mPT via loss of OSCP similarly turns the mitochondrial unfolded protein response from a classically positive force in longevity to lifespan shortening [134]. These observations suggest that mPT may both directly and indirectly modulate aging by governing whether stress defenses such as autophagy or the unfolded protein response promote longevity or shorten lifespan. To our knowledge these are among the first data to implicate a potential causal role for the mPT in normal aging, rather than associations of enhanced mPT with aging and degenerative and aging-associated diseases [110, 111, 275–278].

However, investigation of the role of the mPTP in aging is far from complete. Challenges arise not only surrounding the uncertain protein identification of the mPTP and its regulatory components, but also from the difficulty of directly observing mitochondrial permeability in vivo during aging. Age-dependent physiological triggers of the mPT must be identified and investigated in order to drive the field forward. Further, it will be important to establish how these triggers signal through various mPTP regulatory components to modulate mitochondrial membrane integrity. To enable these advances, will not only require the tools to modulate functionality of mPTP regulatory components in vivo across the lifespan, but also more sophisticated means of visualizing mPT in situ in aging in response to physiologically relevant stimuli and during the normal aging process.

## Conclusion

The mitochondrial permeability transition was first observed in the mid twentieth century, but identification of its core molecular components and their regulatory partners continues to prove elusive. Nevertheless, genetic knockout experiments provide good evidence for regulatory and/or structural roles of the ANT, F-ATP synthase and CypD. Furthermore, progress has been made in recording channel activity from purified versions of several putative pore-containing candidates. The recordings of reconstituted candidate complexes including F-ATP synthase monomers and dimers and ANT have demonstrated sensitivity to reagents that regulate mPT, suggesting that they contain authentic pore proteins. In addition, reconstituted purified F-ATP synthase c-subunit ring has been demonstrated to form a channel. Problems that have arisen in determining an atomic structure for the actual pore within the F-ATP synthasome protein complex include difficulty in demonstrating the intactness of the protein complex after detergent solubilization and difficulty in correlating the in vitro structure that lacks a physiological cell and membrane regulatory milieu with the in vivo structure. Remaining issues in the field include defining whether there is more than one protein type of mPT pore and if so, determining how different pores work together or possibly interact directly. There may well be other protein and lipid regulators yet to be identified in addition to less highly expressed pore-forming proteins than have been described thus far. The fickle nature of mPT regulation is intriguing in that sensitivity to Ca^2+^ and other putative ligands appears to be variable and of low potency, suggesting that mPT regulatory sites may be on proteins or phospholipids peripherally bound to the pore but not contained directly within the pore itself. The complex multi-protein structure surrounding the pore may act as an inhibiting barrier to mPT, requiring large conformational changes of the actual pore protein, associated regulatory proteins or mitochondrial morphology (e.g., contact sites between the inner and out membrane) to initiate channel activation. Questions remain regarding localization of putative pore proteins near MCU, VDAC and other ion channels and transporters as well as interactions with the mitochondrial fission/fusion machinery, the Bcl-2-family members, hexokinase and cristae morphology. Furthermore, there continue to be uncertainties as to the timing and regulation of the mPTP in ischemia reperfusion-induced injury and in normal aging that are still being actively investigated. Physiological functions for a large conductance inner mitochondrial membrane channel such as the mPTP are clearly relevant to cell Ca^2+^ signaling and in the regulation of cell metabolism. Despite advances, the field of pharmacological regulation of mPTP remains in its infancy. There is much more to be discovered about this fascinating mPTP complex that comprises a potential nexus between cell life and death.
